# mTOR inhibitor introduce disitamab vedotin (RC48-ADC) rechallenge microtubule-chemotherapy resistance in HER2-low MBC patients with PI3K mutation

**DOI:** 10.3389/fonc.2024.1312634

**Published:** 2024-01-25

**Authors:** Ye Hu, Fengxi Chen, Siwen Sun, Lingzhi Xv, Xueqing Wang, Meiling Wang, Shanshan Zhao, Zuowei Zhao, Man Li

**Affiliations:** Department of Oncology & Breast Surgery, The Second Hospital of Dalian Medical University, Dalian, China

**Keywords:** HER2-low metastatic breast cancer, disitamab vedotin (RC48), microtubule targeting agents (MTAs), rechallenge, mTOR inhibitor, PIK3CA mutation

## Abstract

This study aimed to explore the efficacy and potential mechanisms of rechallenge therapy with microtubule-targeting agents (MTAs) in patients with HER2-low metastatic breast cancer (MBC). We performed a systematic review to investigate the rechallenge treatment concept in the field of HER2-low MBC treatment and utilized a series of cases identified in the literature to illustrate the concept. Here we reported two clinical cases of HER2-low MBC patients whose disease progressed after prior treatment with MTAs such as docetaxel and vincristine. When rechallenged with disitamab vedotin ((RC48-antibody-drug conjugate (ADC), a monomethyl auristatin (MMAE) MTA)), both patients achieved a partial response and the final progression-free survival (PFS) was 13.5 and 9 months, respectively. Genomic profiling detected a PIK3CA H1047R mutation in the patients. The patients were treated with everolimus before being rechallenged with RC48, which may lead to a better response. This study further summarizes and analyzes the potential mechanism of the PI3K-AKT signaling pathway in MTA resistance and reveals that the PIK3CA H1047R mutation may be a potential molecular marker for the efficacy prediction of mTOR inhibitors, providing new insights and potential therapeutic strategies for the application of MTAs to MBC patients.

## 1 Introduction

Breast cancer (BC) is the most commonly diagnosed malignancy and has the second highest mortality rate globally among all cancers in women (1). Compared with other tumors, the slowing decline in mortality and the rising incidence of breast cancers are concerning (2). For metastatic breast cancer (MBC), therapeutic goals are prolonging life and symptom palliation. MBC remains incurable in virtually all affected patients (3), and a major contributor to this is the abrogation of the efficacy of chemotherapy owing to the emergence of drug resistance (4, 5). Consequently, additional targeted cancer control interventions are needed. Approximately 60% of human epidermal growth factor receptor 2 (HER2)-negative MBC express low levels of HER2 (HER2-low), defined as a score of 1+ on immunohistochemical (IHC) analysis or as an IHC score of 2+ with a negative result of fluorescence *in situ* hybridization (FISH). These patients have limited treatment options after progression during primary therapy and most commonly receive single-agent palliative chemotherapy. Multiple studies have shown that the objective response rate (ORR) is only 6%–17%, and the median PFS is only 1.6–2.8 months in patients with HER2-negative (including HER2 0, HER2 1+, and HER2 2+) MBC who received more than three lines of chemotherapy (6, 7).

MTAs, such as taxanes, have been a mainstay of BC therapy for decades. However, in patients who have previously used taxanes, the prognosis when reusing taxanes in the first line of relapse or metastasis within 2 years is poor. Furthermore, there are clinical and resistance issues that limit the efficacy of these drugs and pose a challenge to improving patient outcomes. In addition to switching to new treatments, rechallenge has attracted widespread attention. Rechallenge therapy is the reintroduction of the same therapy to a patient who has developed resistance after an interval of treatment (8).

Recent advances in ADC agents have led to the widespread clinical use of drugs that target cancer cells via specific antigens (e.g., HER2 (9–11)). The effectiveness of ADCs in prolonging the survival of MTA-resistant patients has attracted our attention. Trastuzumab deruxtecan (also known as T-DXd and DS-8201) is an ADC that has achieved great success in the treatment of HER2-positive BC. The DESTINY-Breast03 trial showed the superiority of DS-8201 in reducing the risk of progression or death in patients with HER2-positive MBC who had been previously treated with trastuzumab and taxane (12, 13). One of the reasons for the success is that DS-8201 consists of a topoisomerase I inhibitor as a payload, which does not develop cross-resistance with MTAs and has an effective bystander effect. RC48 consists of MMAE as a payload (14). MMAE exhibits a marked mitotic inhibitory effect by inhibiting tubulin polymerization (15, 16). Pre-clinical studies and two clinical studies revealed that RC48 demonstrated consistent efficacy in HER2-positive and HER2-low advanced BC (17–19). In 2021, the American Society of Clinical Oncology (ASCO) announced that RC48 can achieve good efficacy in patients with HER2-positive or HER2-low advanced BC who had received multiline treatments, with a median PFS of 4.0 months in the HER2-positive and 5.7 months in the HER2-low (20). Further studies are needed to explore how MMAE-ADC agents are more effective at prolonging the prognosis of patients with HER2-low advanced BC compared with those with HER2-positive advanced BC.

As mentioned above, the role of rechallenge therapy in third-line or fourth-line settings is not clear, but rechallenge could be a possibility for patients who do not have any other valid treatment options (8, 21). Treatment may cause epigenetic alterations that induce chemoresistance, but altered therapy or intermittent therapy may restore the epigenetic features. On this basis, our study explored the rechallenge treatment concept in the field of HER2-negative MBC treatment and used a series of cases to illustrate. Furthermore, we suggest a new strategy that rapamycin (mTOR) inhibitors can be used as an intermittent therapy to prolong PFS in patients with HER2-negative MBC who receive MMAE-ADC agents after MTA progression, and the PIK3CA H1047R mutation may be a potential molecular marker.

## 2 Materials and methods

### 2.1 Search strategy

An electronic search was conducted using PubMed. The following search terms were used: breast cancer, HER2, rechallenge, microtubule-targeting agent, mTOR inhibitors, PI3K (phosphoinositide 3-kinase)-AKT pathway, PIK3CA mutation. The titles and abstracts of all remaining citations were reviewed, and irrelevant citations were discarded. The full text of potentially relevant studies was retrieved and evaluated. A manual search of the reference list of relevant reports was performed to identify any relevant studies missed by the search strategy. The evaluation of efficacy was evaluated using the Response Evaluation Criteria in Solid Tumors (RECIST) guidelines (version 1.1).

## 3 Results

**Figure 1:**
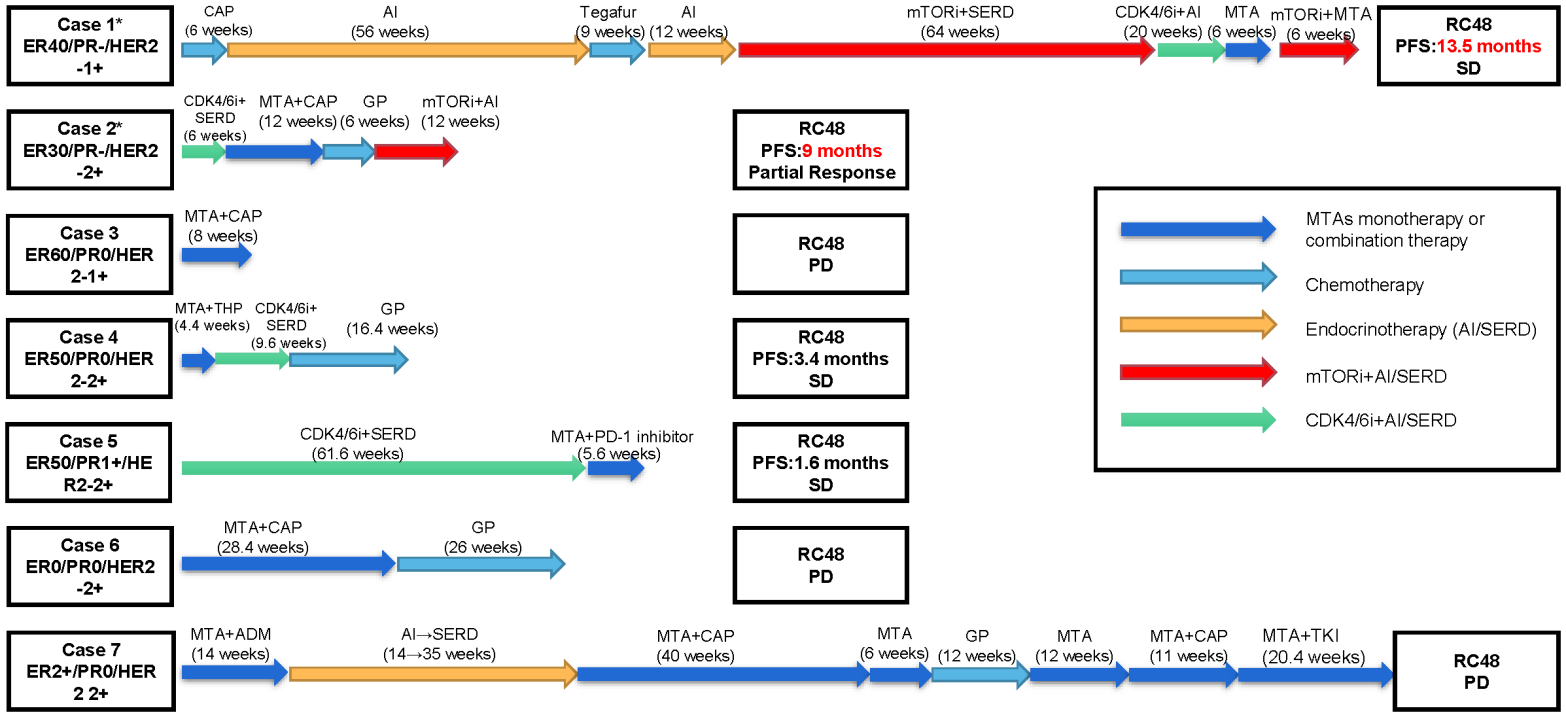
Patients’ overview. Clinical timelines for the seven cases of metastatic breast cancer patients in which RC48 was reintroduced after the progression of multiline standard-of-care microtubule inhibitors. Patients’ histories are shown from advanced first-line treatment until RC48 reintroduction. The arrows represent distinct therapies and durations. The asterisk represents patients carrying PIK3CA H1047R mutation. ER, estrogen receptor; PR, progesterone receptor; HER2, human epidermal growth factor receptor 2; SD, stable disease; PD, progressive disease; CAP, capecitabine; GP, gemcitabine + cisplatin/carboplatin; THP, docetaxel + trastuzumab + pertuzumab; ADM, doxorubicin; MTA, microtubule-targeting agent; AI, aromatase inhibitor; SERD, selective estrogen receptor downregulator; mTORi, mammalian target of rapamycin inhibitors; CDK4/6i, cyclin 4- and 6-dependent kinase inhibitors; PD-1 inhibitor, programmed cell death 1 inhibitor; TKI, tyrosine kinase inhibitor.

**Figure 2:**
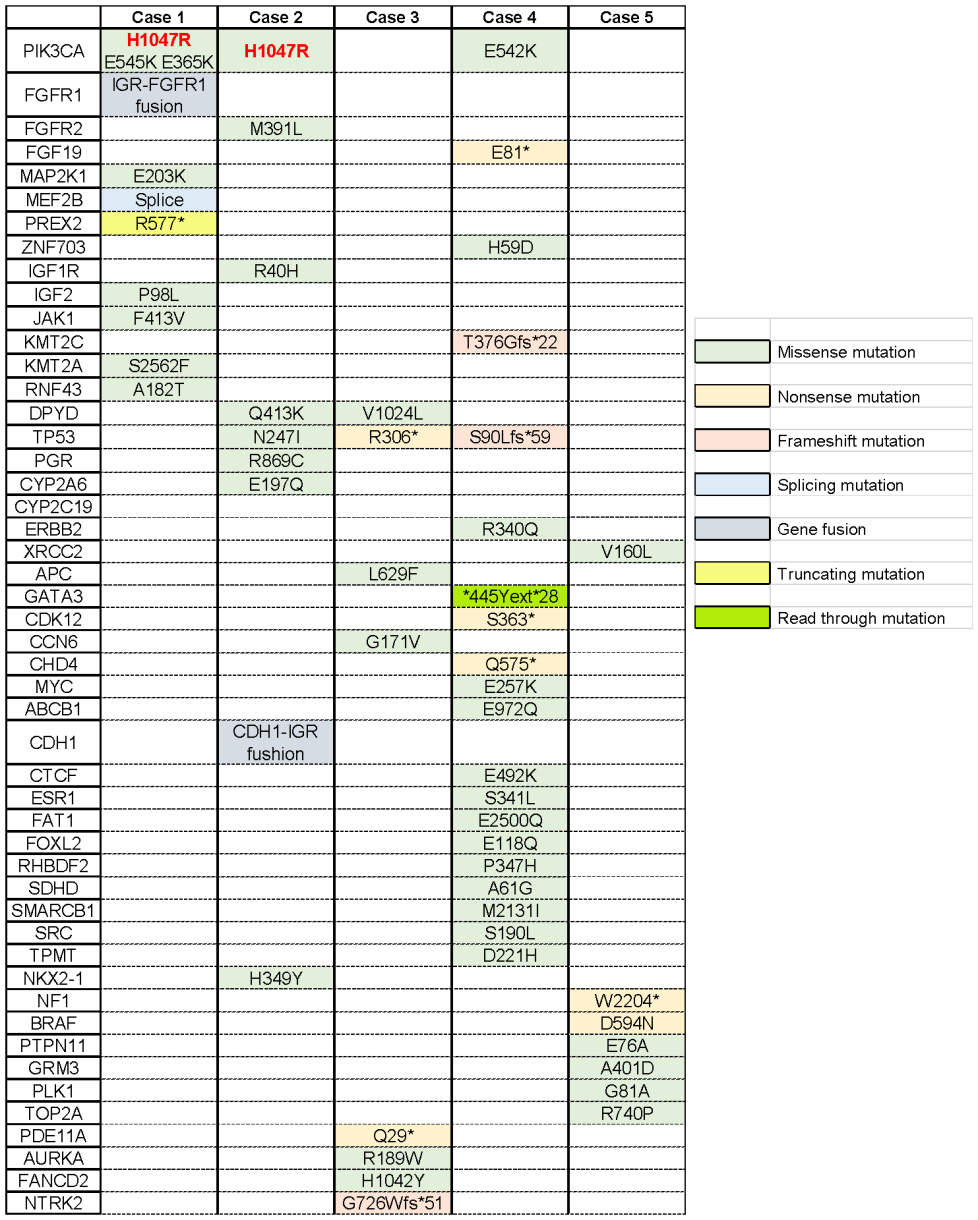
Mutational profiling of breast cancer cases. Single-nucleotide variants are depicted across the five metastatic breast cancer patients. The asterisk indicates that this site mutates into a terminator so that the stop codon is encoded in advance, producing a truncated protein.

We reported a series of cases of MBC patients in which RC48 was reintroduced after the progression of multiline standard-of-care microtubule inhibitors as interventional therapy with mTOR inhibitors and achieved good PFS (**Figure 1**). Additionally, five cases of MBC patients in MTA rechallenge therapy had undergone next-generation sequencing (NGS), and the mutational signature profiling is shown in **Figure 2**.

### 3.1 Case series: five failed cases in HER2-low MBC

Five patients (cases 3–7), who were diagnosed with HER2-low MBC, received multiline chemotherapy treatment, including taxanes, vinorelbine, etc. However, when these patients were given RC48 directly after MTA treatment, the disease developed within 1 or 2 months (**Figure 1**).

### 3.2 Case 1: a 58-year-old woman with HER2-low MBC

**Figure 3:**
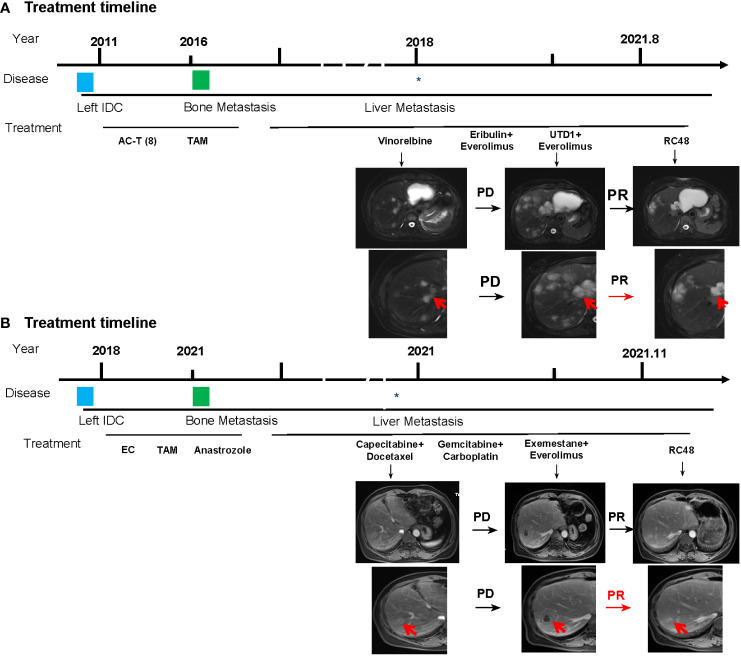
Basic information of the patients. **(A)** Treatment timelines and images of liver metastases of the patient in case 1. **(B)** Treatment timelines and images of liver metastases of the patient in case 2. AC, doxorubicin + cyclophosphamide; T, paclitaxel, TAM, tamoxifen; UTD1, utidelone; RC48, disitamab vedotin. The asterisk indicates when the patient developed liver metastases, and the red arrow represents the tumor tissue.

In May 2011, the patient underwent radical mastectomy for left BC with a pathologic diagnosis of invasive ductal carcinoma of the left breast (pT2N2M0). The IHC analysis confirmed that the patient was estrogen receptor (ER)-negative, progesterone receptor (PR)-negative, and HER2-0. After the operation, adjuvant chemotherapy and radiotherapy and adjuvant endocrine standard therapy were taken in 5 years. However, the patient developed bone metastasis in 2016, which was diagnosed as ER 40%, PR-negative, and HER2 1+. After local radiotherapy and a multiline line of endocrine therapy (ET), the disease was poorly controlled, and a liver malignancy was diagnosed by PET/CT in 2018. Thus, the patient received two cycles of vinorelbine (MTA) chemotherapy. However, the tumor markers increased continuously, accompanied by enlargement of the liver lesions (**Figure 3A**). After the failure of previous treatments, a genomic analysis was performed and the PIK3CA H1047R mutation was identified (**Figure 2**). The patient was started on everolimus plus eribulin/utidelone for 9–10-line therapy. Subsequently, the patient received RC48 for 15 cycles (2 mg/kg, ivgtt, 14 days/cycle), after which her condition stabilized and the PFS reached 13.5 months. This case suggested that mTOR inhibitors as intermittent therapy can reverse the resistance of microtubule inhibitors.

### 3.3 Case 2: a 58-year-old woman with HER2-low MBC

In 2018, the patient received a modified radical mastectomy for left BC. At the time of initial diagnosis, the postoperative pathology was invasive BC (pT1N0M0). The IHC analysis found that the cancer was ER 70%, PR 30%, and HER2-negative. After adjuvant chemotherapy and adjuvant ET, sternal metastasis occurred in February 2021, which was identified as BC metastasis by bone biopsy, and the IHC analysis confirmed that the cancer was ER 30%, PR-negative, HER2 2+, FISH no amplification, and Ki67 30%. Following local radiotherapy and multiline ET, the disease remained poorly controlled and was confirmed as a liver malignancy by PET/CT; consequently, the patient received two cycles of vinorelbine chemotherapy. The patient received advanced first-line ET with abemaciclib plus fulvestrant. Liver metastasis developed after two cycles, and the curative effect was evaluated as progressive disease (PD).

Subsequently, docetaxel (MTA) combined with capecitabine chemotherapy was administered until the liver lesion progressed after four cycles. Genomic profiling was performed and detected a PIK3CA H1047R mutation (**Figure 2**). The liver metastases continued to deteriorate after two cycles of gemcitabine combined with a carboplatin regimen and 1 month of ET with exemestane plus everolimus. However, when the patient received RC48 for four cycles, the intrahepatic lesions significantly reduced in number and size, achieving a partial response (**Figure 3B**). The final PFS was 9 months.

## 4 Discussion

In our study, the failed cases treated with MMAE-ADC agents after the progression of multiline MTA treatment indicated that the efficacy of treatment may be affected if the chemotherapy drug is the same as ADC-payload. This is mainly because they have the same anti-tumor effect, and the patients develop resistance after the progression of chemotherapy. In contrast to these cases, we identified two BC patients who received RC48 after MTA progression and achieved a better prognosis. These two patients had the PIK3CA H1047R mutation detected by genomic profiling and were treated with everolimus before starting the RC48 treatment. The PFS of these two patients treated with RC48 was 13.5 and 9 months, respectively. In addition, one patient achieved a continuous partial response after treatment with RC48. These two cases suggested that mTOR inhibitors as an interval treatment can promote the efficacy of MMAE-ADC agents after the progression of multiline MTA treatment in HER2-low BC. Meanwhile, this study provided new insights into the challenges faced by microtubule inhibitors, implying that mTOR inhibitors can be used as inducers to restore the therapeutic effects of microtubule inhibitors. We hypothesize that mTOR inhibitors as inducers can effectively enhance the anti-tumor effect of MTAs in BC with HER2-low expression.

MTAs are widely used in clinical practice, and numerous studies have identified resistance to paclitaxel and docetaxel. The major structural subunit of microtubules is tubulin, which is composed of α- and β-tubulin monomers that form a dimer and assemble onto the positive ends of the growing microtubule. In many cancer cell lines, MTA resistance results from alterations in microtubule dynamics and binding sites on the microtubules (22, 23). The mechanism of MTAs can be divided into three aspects: (1) disturb the spindle assembly: MTAs, such as taxol, impair the assembly/disassembly dynamic balance of microtubule, thereby activating the spindle assembly checkpoint (SAC) through unattached kinetochores (24); (2) influence on microtubule dynamics: The altered expression of microtubule proteins causes mitotic spindle abnormalities and cell cycle arrest, ultimately leading to genomic instability, which is considered a hallmark of a range of cancers (25–27). Analysis of clinical specimens has shown that, in many cancers, a high expression of several β-microtubulin isoforms is associated with aggressive clinical manifestations, chemotherapy resistance, and poor patient prognosis in several cancers, including breast, colon, and renal cancers (28, 29); and (3) lead to mitotic arrest: The surface of the globular portion of microtubulin contains several pockets that act as intercalation sites for MTAs, thereby affecting the structure of microtubulin. Several studies have demonstrated that treatment with MTAs with this property leads to mitotic arrest and thus cell death (30, 31). The emergence of compensatory pathways leads to MAT resistance due to the alteration of some molecular features during tumor development. Restoring these resistance alterations will re-sensitize the cells to MTAs (32). In addition to our report, other studies have reported a relationship between the PI3K-AKT pathway and MTAs. This pathway is a vital oncogenic pathway and can induce tumor cell survival, proliferation, and metastasis (33–35). Several studies have demonstrated that low-dose PI3K-AKT inhibitors are effective in reversing paclitaxel resistance in different tumor types, including gastric, ovarian, lung, and prostate cancers (36–39). We further explored the mechanisms associated with these microtubule-targeted drug re-sensitization.

**Figure 4:**
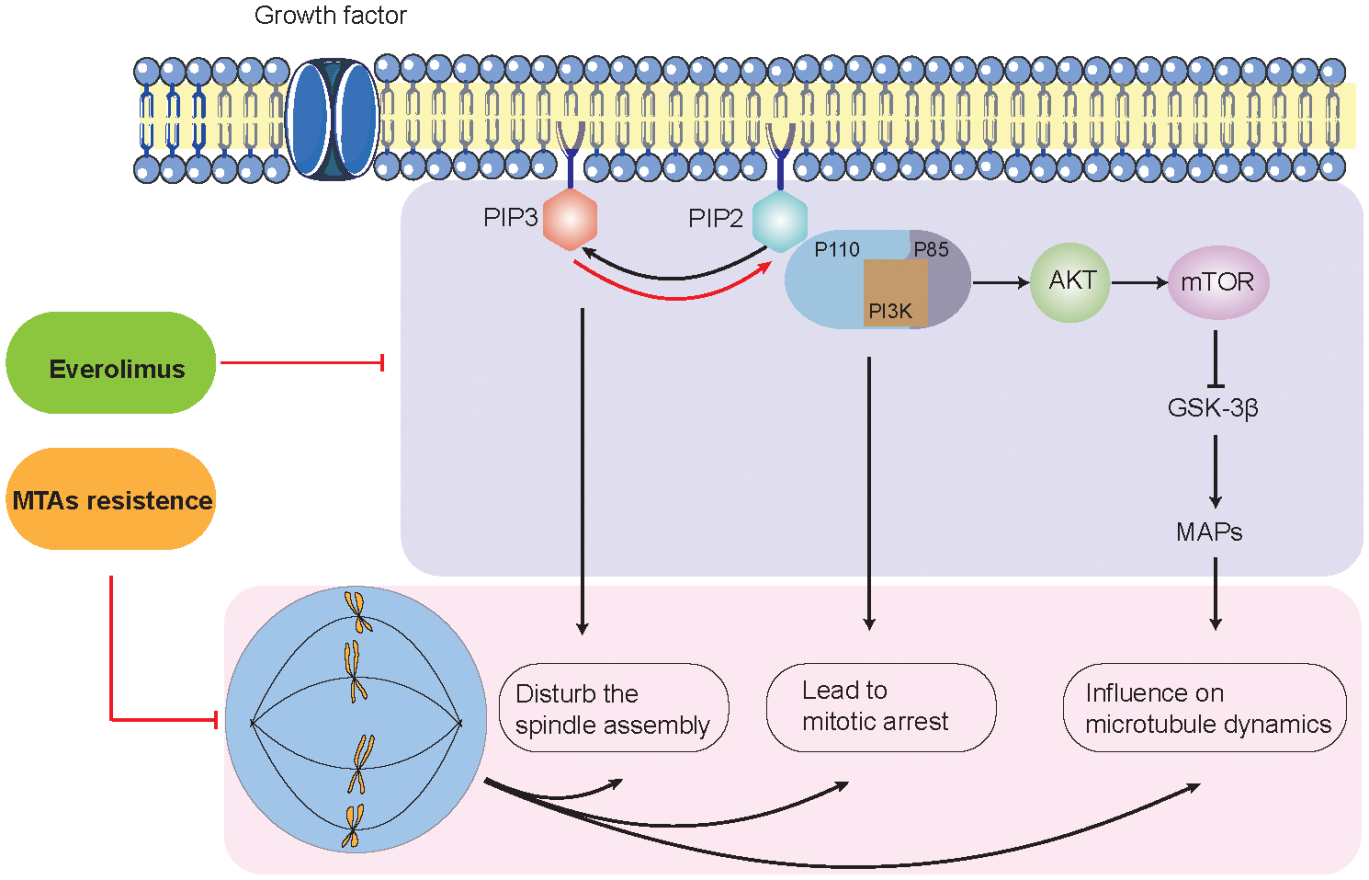
Potential molecular mechanisms between PI3K/AKT signaling pathway and microtubule-targeting agent resistance.

As we all know, tumor cells are often accompanied by abnormally active mitosis and disruption of spindle dynamics (40, 41). Many microtubules and related proteins consist of the basic structure of the mitotic spindle and are involved in the regulation of mitotic spindle dynamics (42). MTAs inhibit cancer cell proliferation by disrupting the mitotic spindle (43, 44). It has been demonstrated that the activation of the PI3K-AKT signaling pathway is closely related to the stability of the microtubules and the mitotic spindle. Gris–Oliver and colleagues suggested that 64% of HER2-negative BC xenografts with resistance to eribulin (microtubule inhibitors) show PIK3CA, PIK3R1, or AKT1 mutation, indicating that PI3K pathway activation may induce resistance or early adaptation to microtubule inhibitors (45). According to the literature, we explored the potential molecular mechanisms by which mTOR inhibitors reverse MTA resistance by inhibiting the PI3K-AKT signaling pathway (**Figure 4**).

On the one hand, the interaction between PI3K-AKT mainly involves two metabolites: PIP2 (phosphatidylinositol-4,5-bisphosphate) and PIP3 (phosphatidylinositol-3,4,5-bisphosphate) (46, 47). PI3K is an intracellular phosphatidylinositol kinase. PI3K consists of a regulatory subunit (p85) and a catalytic subunit (p110) (48). When the ligand binds to the membrane receptor, the receptor activates p85 and recruits p110, which, in turn, catalyzes the production of PIP3 from PIP2 on the inner surface of the membrane (49–51). p110α is activated at the initiation of mitosis, producing PIP3 in the mesocortex of metaphase cells to guarantee proper orientation of the spindle. Lipid phosphatases (e.g., PTEN) can metabolize (dephosphorylate) PIP3 back to PIP2, thereby terminating the PI3K signaling pathway. In addition, PI3K-C2α, a class II PI3K family member, interacts with the transformed acidic coiled-coil 3 (TACC3) and clathrin heavy chain (CHC) complexes to stabilize the kinetochore–microtubules formed by the spindle (52, 53). Due to the critical role of PI3K-AKT signaling in spindle formation, drug inhibitors such as LY-294002 and MK-2206 can lead to aberrant centrosome and mitotic spindle formation, thereby reversing MTA resistance and enhancing antitumor effects (52, 54, 55).

On the other hand, the PI3K-AKT-mTOR pathway has been implicated in the regulation of microtubule stability (35, 56–58). Onishi et al. showed that the addition of the pan-PI3K inhibitor LY-294002 destabilized microtubules in fibroblasts (59). Previous studies have shown that the localization of AKT to microtubules is important for sustaining AKT phosphorylation. Consistently, the introduction of a predominantly negative form of AKT into cells showed destabilized microtubules (57, 58). The regulation of microtubule dynamics by the PI3K-AKT pathway can be transduced by glycogen synthase kinase-3β (GSK-3β). As previously mentioned, PI3K-AKT inhibits the activity of GSK-3β activity (60). The inhibition of PI3K-AKT signaling by LY-294002 induces the activation of GSK-3β, which, in turn, regulates a large repertoire of protein substrates, including microtubule-associated proteins (MAPs) (60). MAPs interact with tubulin dimers of the microtubule (e.g., MAP1, MAP2, MAP3, MAP4, and tau) (61). This interaction leads to microtubule stabilization and subsequent polymerization—for example, MAP4 decorates the microtubule network and participates in microtubule assembly and endosomal vesicle trafficking along microtubule tracks. Thapa et al. discovered that MAP4 is a binding partner of the p110α catalytic subunit of PI3Kα (62). The loss of MAP4 perturbs PI3Kα recruitment along microtubule tracks and endosomes and disrupts PI3Kα association with activated receptor complexes, which collectively contribute to impaired PI3Kα activation (62–64). A previous study revealed that LY-294002 did not induce cell death but resulted in the marked and selective enhancement of the induction of apoptosis by microtubule-destabilizing agents such as vincristine (60). The mechanism is that blockade of the PI3K-AKT pathway induces the activation of GSK-3β, which phosphorylates MAPs such as tau and thereby reduces their ability to bind and stabilize microtubules (64).

Previous studies suggested that cleaved-PARP may be involved in the action of the PI3K-AKT inhibitors in the paclitaxel-resistant cell line (65, 66). PARP is a family of enzymes of poly (ADP-ribose) polymerases. In addition to mediating DNA damage response pathways, PARP is crucial in regulating genome stability through transcription and regulating the expression of oncogenes and tumor suppressor genes (67, 68). In the cases in this study, the administration of the combination of everolimus and ET or chemotherapy did not improve PFS and disease progression continued. However, after everolimus treatment, patients with HER2-negative BC who received RC48 achieved an improvement in PFS. These results indicate that mTOR inhibitors may act as inducers that support MMAE-ADC agent rechallenge in patients with MTA-resistant HER2-negative BC. HER2 and insulin receptor substrate (IRS) stimulate the RAS-ERK-RSK and AKT-mTOR pathways. Activation of mTOR signaling phosphorylates S6 kinase 1 (S6K) and eukaryotic initiation factor 4E-binding protein 1 (4E-BP1), which promote cancer growth and metastasis (69). The use of mTOR inhibitors and PI3K inhibitors to overcome resistance to current HER2 therapies is an active area of research (70). These may be the reasons for the re-sensitization of MTAs by mTOR inhibitors, and we still need more basic and clinical trials to verify this.

PIK3CA mutations are present in approximately 20% of HER2-positive BCs (71, 72). In patients with advanced hormone receptors (HR)+/HER2-negative BC, 28% of PIK3CA mutations were identified in circulating tumor DNA (73, 74). The results from the SAFIR02 trial showed that PIK3CA mutations were associated with chemoresistance and poorer overall survival (OS) in ER+ HER2-negative BC (75). PIK3CA was the most frequent mutation observed, associated with an increased kinase activity of the PI3K pathway. In BC, the most frequent PIK3CA mutations lead to protein residue changes at the following positions of exon mutations: E542K (76–78), E545K (77, 78) in exon 9 and H1047R (77, 78) in exon 20, comprising approximately 78% of all PIK3CA mutations observed in breast tumors (79). Numerous studies have demonstrated that these hotspot mutations prompt transformation and tumorigenicity by inducing enhanced PI3K function and activating downstream signaling in the AKT-mTOR pathway (75, 80), with robust *in vitro* and *in vivo* transformation phenotypes (81–84). Different mutations at the same residues had different phenotypic activities depending on their mutation frequency. It has been reported that a higher frequency mutation (H1047R) showed a stronger tumorigenic phenotype compared to mutations of E542K, E536K, and E545K (85–88). In recent years, the relationship between gene mutations and response to mTOR inhibitors has received particular attention. Consistently, experimental studies have demonstrated that BCs with PIK3CA mutations are more sensitive to everolimus, and the IC50 value of the mTOR inhibitor is lower for H1047R than for E542K or E545K (89). The PIK3CA H1047R mutation was also reported to confer sensitivity to everolimus in early-phase clinical trials in many types of cancers (90, 91). The potential underlying mechanism is that the PIK3CA H1047R mutation is a stronger driver of tumor development than other types of PIK3CA mutations (92, 93).

Therefore, patients with PIK3CA H1047R mutations were more sensitive to the mTOR inhibitor everolimus (94). Based on the previous studies, among MBC patients who had been treated with everolimus, patients with the PIK3CA H1047R mutation had longer PFS compared with patients with wild-type or other mutant forms of PIK3CA (*p* <0.050) (95). It is worth noting that PIK3CA H1047R was detected in two cases with a favorable prognosis that was mentioned above (**Figure 2**). Both of them used mTOR inhibitors before MTA rechallenge treatment and showed longer PFS.

In summary, different mutations in PIK3CA lead to different sensitivities of tumors to everolimus, and patients with PIK3CA H1047R mutations were more sensitive to the mTOR inhibitor everolimus. This may be because the PIK3CA H1047R mutation is a stronger driver of tumor development than other types of PIK3CA mutations (94). More importantly, relevant clinical studies suggested that PIK3CA H1047R mutations may be a potent biomarker of sensitivity to everolimus in MBC (95–97). The combination of alpelisib, a selective PIK3CA inhibitor, with mTOR inhibitors has also shown a synergistic efficacy in PIK3CA-mutated (H1047R) hepatocellular carcinoma (98). These results still need further exploration in clinical trials.

In our study, we found that patients had other mutations in addition to PIK3CA. However, they were not associated with mTOR inhibitors and MTAs—for example, mutations in the fibroblast growth factor receptor (FGFR) 1/2 gene were associated with FGFR kinase inhibitor resistance (99, 100). Dihydropyrimidine dehydrogenase genotyping is related to the sensitivity of fluoropyrimidines (5-fluorouracil, capecitabine, and tegafur) (99, 101). Sensitivity to combined RAF kinases (BRAF) and mitogen-activated protein kinase kinase (MEK) treatments is associated with co-mutations of mitogen-activated protein kinase kinase 1 (MAP2K1) and BRAF (102). There are still some meaningful variations that are worth exploring. In addition, tumor progression is, in essence, an evolutionary process, and different adaptive changes in molecular characteristics during tumor development can enable tumors to progress through tumor pathologic stages or transformation barriers (40, 103). According to the theory of “evolutionary selection”, molecular pathways that are activated or inactivated during tumor evolution may play important adaptive roles in promoting tumorigenesis, proliferation, and metastasis. This evolutionary theory could explain the molecular mechanisms that emerged during tumor evolution in the patient in our case after mTOR inhibitor treatment, and restoration of these alterations would re-sensitize cells in HER2-low MBC to MTAs.

Two-thirds of BC patients express HR and lack HER2 overexpression and/or amplification (104, 105). For these patients, the combination of ET with cyclin-dependent kinase (CDK)4/6 inhibitors exhibited significant survival benefits and is now the gold standard for HR+/HER2-negative MBC (106, 107). The choice of drug for use in clinical practice should be based on the response to prior treatment before progression. Previous studies revealed that when the disease progressed after the administration of microtubule inhibitors, rechallenging with the same drugs seems to be inadvisable. Conversely, few clinical studies have evaluated the role of rechallenge in BC (108, 109). We summarized eight reports about rechallenges in BC (**Table 1**), which highlight the clinical importance of paying more attention to rechallenges. Toulmonde et al. demonstrated docetaxel rechallenge as a second, third, fourth, or more line of chemotherapy in the metastatic setting. Among the 33 patients with disease assessed according to RECIST guidelines, 14 (42.5%) had a partial response and 11 (33.5%) had a stable disease >6 weeks. Globally, 55 patients (76%) obtained a benefit from the treatment. The median time to progress and OS were 5.7 months (95% CI: 5.0–6.3) and 10.2 months (95% CI: 8.6–11.8). This retrospective analysis supported the pragmatic strategy to retreat patients with MBC with docetaxel (111). Meanwhile, some trials with trastuzumab and CDK inhibitors have demonstrated the potential of rechallenge in clinical applications (113, 114, 116). In recent years, there have been many studies on ADC drugs in BC with HER2-low-expression, including a number of clinical trials (**Table 2**).

**Table 1:** Prior reports about rechallenge in breast cancer.

Regimen	No. of patients	Results	Subtype	References
Tamoxifen	MCF7 vs. MCF7-TAM12.5	Metastatic potential decreased;proliferation and clonogenicity increased	p-AuroraA/B upregulated	(110)
Docetaxel rechallenge	72	55 patients (76%) obtained a benefit from the treatment	MBC	(111)
Adjuvant anthracyclines vs. anthracycline rechallenge	70	ORR: 38%; CB: 71%; mOS: 16.5	MBC	(112)
(Neo) adjuvant trastuzumab–trastuzumab rechallenge vs. lapatinib	101 vs. 27	No significance between two groups	HER2+	(113)
Lapatinib-resistant vs. trastuzumab Trastuzumab-resistant vs. lapatinib rechallenge	74 50	CB: 32%	HER2+	(114)
Pembrolizumab vs. atezolizumab rechallenge	1	Excellent response without drug-related adverse effects	TNBC	(115)
CDK inhibitors	6	1 CR 2 PFS, 6 months 1 PFS, 10 months	HER2-negative MBC	(116)
Anthracyclines and taxanes vs. eribulin rechallenge	22	No significance in OS between two groups	MBC	(117)

ORR, objective response rate; CB, clinical benefit; mOS, median overall survival; PFS, progression-free survival.

**Table 2:** Clinical trials of antibody–drug conjugate (ADC) drugs in breast cancer with HER2-low expression.

Target	ADC drugs	Tumors	Status	Combination	Trial phase	Results	NCT
HER2	DS8201	Third-line advanced HER2-low BC, post-chemo	Active, not recruiting	None	Phase III	PFS: 10.1 m OS: 23.9 m mPFS: 9.9 m	NCT03734029 (118)
		Third-line advanced HER2-low BC, chemo-naive	Recruiting	None	Phase III		NCT04494425
	SYD985	Neoadjuvant HER2-low BC		None	Phase II		NCT01042379 (119)
	ARX788	R/R Advanced HER2-low BC		None	Phase II		NCT05018676
	RC48	R/R Advanced HER2-low BC		None	Phase III		NCT04400695
	MRG002	R/R Advanced HER2+ BC		None	Phase II		NCT04924699
		R/R Advanced HER2 low BC		None	Phase II		NCT04742153
	A166	R/R Advanced HER2-expressing cancer		None	Phase II		NCT03602079
		HER2-low advanced breast cancer		DS-8201a (single arm)		ORR: 37.0% 95%CI: 24.3%–51.3%	
Trop-2	DS-1062	Second-line advanced hormone receptor+/HER2- BC		None	Phase III		NCT05104866
	IMMUN-132	Third-line advanced hormone receptor+/HER2- BC		None	Phase III	ORR: 31.0% DOR: 7.4 m 95%CI: 4.4%–18.3% PFS: 6.8 m 95%CI: 4.6%–8.9%	NCT03901339 (120)
		Post-neoadjuvant HER2- BC		None	Phase III		NCT04595565 (121)
CD166	CX-2009	Post-neoadjuvant HER2- BC		None	Phase III		NCT04595565 (121)
HER3	U3-1402	Preoperative hormone receptor+/HER2- BC	Recruiting	None	Phase I	The primary endpoint is a CelTIL score after one single dose	NCT04610528 (122)

PFS, progression-free survival; mPFS, median progression free survival; OS, overall survival; ORR, objective response rate; DOR, duration of response; m, month.

However, most clinical trials are still recruiting and further research results are anticipated. RC48 is the representative MMAE-ADC agent for BC treatment, showing consistent efficacy in posterior line HER2-low-expressing MBC. Collectively, the role of rechallenge therapy in the treatment of MBC has not yet been established—whether a MMAE-ADC agent is feasible as a choice after MTA resistance rechallenge in BC. Further studies are needed to verify this strategy.

In conclusion, our study provides a new insight that mTOR inhibitors, as an intermittent treatment, may induce sensitivity to MMAE-ADC agents in HER2-low-expressing BC, especially after disease progression following MTA multiline treatment. The NGS indicates that PIK3CA H1047R mutation may correlate to this better prognosis. Our data may help clinicians and patients make personalized decisions to try and rechallenge the ominous situation of metastatic disease to maximize efficacy and extend patient wellbeing for as long as possible.

## Author contributions

YH: Data curation, Writing – original draft, Writing – review & editing. FC: Data curation, Writing – original draft, Writing – review & editing. SS: Data curation, Writing – original draft, Writing – review & editing. LX: Methodology, Supervision, Writing – review & editing. XW: Supervision, Validation, Writing – review & editing. MW: Supervision, Validation, Writing – review & editing. SZ: Investigation, Visualization, Writing – review & editing. ZZ: Conceptualization, Funding acquisition, Writing – review & editing. ML: Conceptualization, Funding acquisition, Writing – review & editing.
